# In patient stroke rehabilitation efficiency: Influence of organization of service delivery and staff numbers

**DOI:** 10.1186/1472-6963-8-86

**Published:** 2008-04-17

**Authors:** Jean Woo, Shiu Yu Chan, Mi Wan Cecilia Sum, Eric Wong, Yeuk Ping Maria Chui

**Affiliations:** 1 Department of Medicine and Therapeutics, The Chinese University of Hong Kong, Shatin, NT, Hong Kong SAR, China; 2 Shatin Hospital, Shatin, NT, Hong Kong SAR, China; 3 School of Public Health, The Chinese University of Hong Kong,, Shatin, NT, Hong Kong SAR, China

## Abstract

**Background:**

Outcomes of inpatient stroke rehabilitation need to be reviewed in terms of optimal resource utilization (staff time, service organization, and duration of stay). We compared FIM efficiency scores between three hospitals, and also variation in FIM scores over a ten year period in one hospital undergoing reduction in staff numbers, to examine the relationship between outcome and service characteristics.

**Method:**

This is a retrospective study comparing the mean FIM efficiency for stroke patients (FIM score – FIM admission score) divided by duration of stay for 2005 among three rehabilitation hospitals adjusting for age and baseline FIM score, and a longitudinal study of changes in mean FIM efficiency during a ten year period in one hospital, to examine the effects of different service organization and staff numbers.

**Results:**

FIM efficiency (FIMEG) was inversely associated with age, and positively associated with admission FIM score. FIMEG was higher in the hospital with a coordinated care plan involving medical, nursing, occupational, physiotherapy staff and other healthcare providers working as a team, with a seamless interface with community rehabilitation services. Over a ten year period, reduction in staff numbers was associated with reduction in FIMEG, which may be offset to some extent by service re-engineering.

**Conclusion:**

Within hospital organization of stroke rehabilitation services may influence outcome. A critical number of staff may be identified for the provision of services, below which rehabilitation efficiency may be affected.

## Background

Stroke is the second commonest cause for admission to acute hospitals in Hong Kong, accounting for the largest number of bed days occupied (375,000) in all hospitals per year [1]. It also contributes significantly to the disability burden [2], accounting for approximately 50% of the admissions to long term care institutions. Currently patients with stroke are admitted to an acute hospital initially for neuroimaging and other investigations and then transferred to a non-acute hospital for rehabilitation when clinically stable. In these hospitals, rehabilitation programmes may vary in nature, duration of treatment per day, as well as total duration of stay.

The provision of these programmes is to a certain extent empirical, being affected by staff numbers as well as the shortage of hospital beds. In this scenario, for optimal use of health care resources, it would be necessary to identify the most efficient practice in terms of the largest gain in function with the lowest duration of stay. At the same time, recovery should not be curtailed at the expense of lack of rehabilitation resource.

In order to address this issue, the results of rehabilitation programmes need to be quantified. There has been increasing emphasis on the need for standardizing data collection on functional status for the purpose of quality assurance and for setting health and research policies, which should broadly cover an individual's ability to perform activities of daily living and to participate in societal activities.

In the post acute care setting, various outcome instruments have been developed, such as the Functional Independence Measure (FIM) [3,4], the Barthel Index (BI) [5], the Minimum Data Set (MDS) [6], the Outcome and Assessment Information Set for Home Health Care (OASIS) [7], the London Handicap Scale (LHS) [8], and generic quality of life scales such as the Short Form-36 (SF-36) [9] and WHOQOL [10]. A number of these instruments have been validated in Hong Kong, such as the MDS [11], LHS [12], SF-36 [13], and WHOQOL [14]. The MDS is currently used for long term care placement assessment by the Social Welfare Department. As it requires special training and can take over 30 minutes to complete, it has not been used routinely as an outcome measure to monitor outcome of care. The LHS has been used in stroke patients as a research tool to examine handicap for those living at home one year after stroke [15]. Similarly the SF-36 and WHOQOL have largely been used in the community setting to compare individuals with various disease states and normal subjects [16].

The BI is used widely in hospitals to measure progress in rehabilitation, while the FIM is used in addition for initial and pre-discharge assessment in four major non acute rehabilitation hospitals. In comparison with other instruments, the BI and FIM cover a narrower range of functional content across the functional ability continuum, representing the beginning of the continuum, a situation found in the hospital setting. Therefore these two instruments will be most relevant for the evaluation of hospital rehabilitation programmes.

A key requirement of such instruments would be ease of administration and sensitivity to change, both instruments possessing these properties. FIM is a broader assessment compared with BI, covering cognitive and social abilities in addition to other activities of daily living. Therefore it is considered to be more sensitive to change and less limited by floor and ceiling affects compared to the BI. These instruments have been used to predict level of disability on discharge in stroke patients in Hong Kong [17], to assess quality and efficiency [18] as well as cost-effectiveness of rehabilitation services [19], and to determine the type and level of rehabilitation service needs [20].

Currently the Hospital Authority, a government subvented body in charge of providing over 95% of inpatient services in Hong Kong, has been undergoing productivity gain initiatives for a few years. The demand on staff time is constantly increasing, as is the pressure on reducing the duration of hospital stay. It is uncertain how these two factors may affect the provision of rehabilitation services in non-acute hospitals. Therefore it is timely to examine the nature and content of rehabilitation services with respect to quantifiable outcomes in order to optimize resource utilization in terms of staff time and duration of hospital stay.

This study examines variation in stroke rehabilitation outcome using FIM efficiency score among three non acute hospitals with differing service organization, and also variation over a ten year period in one hospital which had been undergoing reduction in staff numbers.

## Methods

This is a retrospective study of the FIM score databases of three non-acute hospitals (A,B,C) containing stroke patient data (age, gender, duration of hospital stay FIM score on admission and discharge). These hospitals are situated in three different areas of Hong Kong, and there are variations in the provision of rehabilitation service that are partly dependent on rehabilitation staff numbers and design of programmes. FIM scoring was used as one of the in service assessment tools, and was carried out by occupational therapists. The maximum total FIM score is 126; the maximum scores for the motor and cognitive components are 91 and 35 respectively.

The FIM efficiency (FIMEG) of the rehabilitation programme was calculated by the gain in FIM (Discharge FIM score – Admission FIM score), divided by the duration of stay. Since patient factors such as age, and baseline level of cognitive/functional/communication impairment affect the progress of rehabilitation, efficiency was adjusted for age and baseline FIM score. The mean efficiency for all stroke patients was calculated for each hospital for the year 2005 and tested for statistical significance between hospitals using analysis of variance (ANOVA), adjusting for age and baseline FIM score. Information regarding the mean duration of treatment per day for patients and the organization of rehabilitation services for stroke patients was collected. The dataset also contained information regarding living arrangement (at home or in long term residential care), and presence of any social support before admission and after discharge. A longitudinal study was also carried out for hospital A analyzing yearly data over a ten year period (1996–2005), during which time there had been steadily declining staff numbers.

### Statistical methods

Spearman's rank correlation was used to examine associations between FIMEG and age and admission FIM score. Chi Square and Kruskal-Wallis tests were used to compare characteristics between hospitals. Analysis of covariance was used to compare the mean FIMEG between hospitals adjusting for age, motor and cognitive FIM score at admission. Bonferroni test was used to correct p values for multiple comparisons. One way ANOVA with polynomial contrast was used to assess the trend of total FIMEG across time, and Bonferroni post hoc multiple comparisons was applied to examine the between year differences. All statistical tests were performed using SPSS for Windows v.14 (SPSS Inc, Chicago, Illinois, USA).

## Results

**Figure 1:**
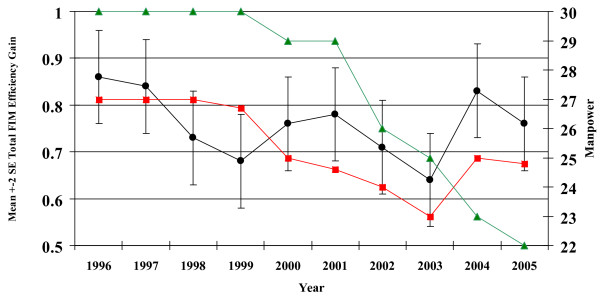
**Mean FIMEG in institution A over 10 years: influence of number of occupational therapists.** nurse numbers (FTE),  OT numbers (FTE),  FIMEG (FTE). Multiple comparisons: 2003<1996 & 1997 & 2004; 1999<1996 (p-values <0.05). a) Remark: Nursing Manpower. • Manpower indicated is for each pair ward which is the same. • 1996 to 1998 – manpower count included 7 pupil nurses in each pair ward. b) 1999 – Service Re-organization. c) 2003 – ward closure.

Apart from the mean age of patients admitted with stroke, there were statistically significant differences in the duration of stay, admission and discharge FIM scores, FIMEG, gender, pre admission and discharge living arrangement, and social support between the three hospitals (Table 1). Within each hospital and for the whole group, the FIMEG was negatively correlated with age and positively associated with admission motor FIM and cognitive FIM scores (Table 2). Adjusting for age of subjects and admission Motor and cognitive FIM scores, there were significant differences in mean FIM efficiency gains between institution A and B, A and C, but no difference between B & C (Table 3). The mean FIM efficiency gain for each year over a ten year period is shown in Fig 1 and Table 4, together with the mean number of occupational therapist and nursing staff for stroke care,  (The number of physiotherapists did not fluctuate during this period). There were significant differences in time trend using ANOVA, and between years using multiple comparisons. The number of occupational therapist and nurses showed a steady decline from 1996 to 2003. The FIM efficiency gain also fell until 1999, when it increased for the next two years and then started falling until 2003, when it again rebounded. In 1999 the rehabilitation service was reorganized. In response to staff reduction, a triaging system was introduced to focus rehabilitation efforts on stroke patients with recovery potential rather than on maintenance therapy. In 2004, the number of occupational therapists was increased.

**Table 1:** Patient characteristics and Functional Independence Measure Score (FIMS) by institution

**Median (25–75percentile)**		**A**	**B**	**C**	**All**
Age (Years)		76 (68–81) (n = 390)	75 (68–82) (n = 600)	75 (68–81) (n = 1220)	75 (68–81) (n = 2210)
Duration of stay (days)		22 (15–32) (n = 390)	25 (17–36) (n = 601)	26 (15–39) (n = 1220)	25 (16–37)* (n = 2211)
Admission FIM score (Total)		71 (42–89) (n = 390)	56 (30–80) (n = 601)	66 (42–81) (n = 1006)	64 (39–86)* (n = 1997)
(Motor)		45 (24–59) (n = 390)	32 (16–50) (n = 601)	43 (25–58) (n = 1007)	40 (22–56)* (n = 1998)
(Cognitive)		25 (16–33) (n = 390)	22 (11–33) (n = 601)	23 (14–29) (n = 1037)	23 (14–31)* (n = 2064)
Discharge FIM score (Total)		91 (57–108) (n = 390)	72 (36–100) (n = 601)	84 (57–105) (n = 1083)	83 (51–105)* (n = 2074)
(Motor)		62 (38–75) (n = 390)	46 (20–68) (n = 601)	59 (38–75) (n = 1160)	57 (31–74)* (n = 2151)
(Cognitive)		29 (19–35) (n = 390)	25 (13–35) (n = 601)	25 (17–31) (n = 1005)	26 (16–33)* (n = 1996)
FIM efficiency gain (Total)		0.64 (0.27–1.07) (n = 390)	0.31 (0–0.72) (n = 601)	0.5 (0.21–0.89) (n = 1004)	0.48 (0.15–0.89) (n = 1995)
(Motor)		0.57 (0.21–0.95) (n = 390)	0.26 (0–0.61) (n = 601)	0.44 (0.17–0.78) (n = 1073)	0.42 (0.11–0.79)* (n = 2064)
(Cognitive)		0 (0–0.15) (n = 390)	0 (0–0.03) (n = 601)	0.04 (0–0.12) (n = 1073)	0.01 (0–0.11) (n = 2064)
No (%)					
Sex	M	206 (52.8%)	364 (60.6%)	627 (51.4%)+	
	F	184 (47.2%)	237 (39.4%)	593 (48.6%)	
Preadmission Living arrangement	Home	355 (91.0%)	477 (79.4%)	1107 (90.9%)+	
	Institution	35 (9.0%)	124 (20.6%)	111 (9.1%)	
Preadmission carer support – alone		50 (12.8%)	93 (15.5%)	189 (15.6%)+	
Preadmission carer support – Family		280 (71.8%)	359 (59.7%)	895 (73.7%)	
Preadmission carer support – Paid help		26 (6.7%)	26 (4.3%)	21 (1.7%)	
Discharge living Arrangement	Home	237 (60.8%)	282 (46.9%)	545 (50.2%)+	
	Institution	153 (39.2%)	319 (53.1%)	531 (49.4%)	
Discharge carer support – alone		9 (2.3%)	14 (2.3%)	41 (3.8%)	
Discharge carer support – Family		189 (48.5%)	249 (41.4%)	496 (46.3%)	
Discharge carer support – Paid help		40 (10.3%)	20 (3.3%)	5 (0.5%)	

Chi Square test between institutions: +P < 0.001

Kruskal-Wallis test between institutions: *P < 0.001

**Table 2:** Spearman's rank correlations with total FIMEG by institutions

	A (n = 375)	B (n = 601)	C (n = 1220)	All Hospitals (n = 2196)
Age of patients (years)	-.292	-.346	-.258	-.283
Total FIM motor score at admission	.420	.449	.532	.494
Total FIM cognitive score at admission	.421	.465	.468	.453

All p-values<0.01

**Table 3:** Comparison between institutions on total Functional Independence Measure Score(FIMS) efficiency gain using analysis of covariance

	Adjusted Mean (SE)*	95%Confidence Interval	Adjusted p-value**	Equality of adjusted means (p-value)***	
Hospital A (n = 375)	.735 (.033)	(.670, .800)	<0.001	A vs C(<0.001)	A vs B(<0.001)
Hospital B (n = 601)	.503 (.027)	(.451, .555)			
Hospital C (n = 1002)	.550 (.020)	(.510, .591)		C vs B (0.488)	

* Adjusted age of patients, total FIMS motor and cognitive scores at admission (all p < 0.001) Using analysis of covariance

** Test for overall equality of adjusted means in the 3 institutions from analysis of covariance

***Adjustment for multiple comparisons using Bonferroni test.

The distribution of total FIM efficiency gain, total FIM motor score at admission and total FIM cognitive score at admission were slightly skewed (skewness statistic were 0.676, -0.371 and 0.275 respectively). As the sample size in each hospital was large enough and the robustness property of the parametric test, ANCOVA is appropriate.

**Table 4:** The mean FIM efficiency gain for each year over a ten year period (also see Fig 1)

**Year**	**No. of patients**	**Year**	**No. of patients**
1996	374	2001	504
1997	311	2002	386
1998	209	2003	345
1999	248	2004	386
2000	428	2005	375

## Discussion

This study shows variation in stroke rehabilitation outcome in terms of FIMEG among hospitals, and also variation with time within one hospital. There is limited information regarding the type of rehabilitation programme in relation to best outcome in the local population, previous studies having been limited to Europe [21,22], the USA and New Zealand [23], and the findings may not be generalized to non-European populations. A marked variation in stroke outcome in seven European countries was observed even when adjusted for case mix and health care resources, and the poor outcome in the UK was partly attributed to differences in the process of care [22]. A comparison between stroke rehabilitation practice and outcome between United States and New Zealand facilities concluded that intensity of therapy may be an important factor. A recent study relating stroke outcome with resource use across 19 countries found a three fold variation in the average number of days of hospital/institutional care (20–60 days), with no relation between health care resource use and outcome in terms of activities of daily living at three months [24]. It was pointed out that differences in treatment pathways and social context seem to be major determinants of resource use, and the study cautioned against use of outcome variables as indicators of quality of stroke care. Our current study examines variation in outcome in the same social and health care system context, such that some conclusions may be drawn regarding service organization of stroke rehabilitation. Furthermore, similar uncertainties exist regarding the duration of therapy [25].

The variation in admission characteristics among the three institutions is a reflection of the differences in geographic region and population characteristics. Compared with the area where institution A is situated, there were more residential care homes in the other areas served by institutions B & C, so that more stroke patients living in long term residential care with more initial disability may be admitted to B or C. This factor may partly explain the lower admission FIM score (both motor and cognitive) and longer duration of stay. Previous studies have also examined the influence of age and admission disability level on recovery and discharge destination. A HK study of 793 Chinese stroke patients identified age, admission disability and impaired cognition as factors predicting stroke disability on discharge [17]. Similarly, a study in Taiwan showed that admission FIM score predicted the degree of functional gain, while age was a factor in predicting FIMEG [26,27]. Even though another study in Hong Kong Chinese pointed out that the percentage gain in FIM score did not vary with age, such that rehabilitation should not be neglected among the very old [28], both admission and discharge FIM scores were negatively correlated with age. Therefore in comparing FIMEG, adjustment needs to be made with respect to age and admission FIMS.

Since efficiency is a measure of two composite factors: gain in function and duration of stay, factors affecting either or both would affect efficiency and hence the variation among the three institutions. A recent review showed that the time taken to achieve best performance ranges from 8–17 weeks depending on stroke severity; summarized the benefits of organized stroke care services and the importance of coordinated care; and discussed the intensity and quantity of therapy [27]. Counter-intuitively quantity did not appear to play a strong role, in that doubling the quantity only resulted in a small increase in functional recovery, a finding compatible with conclusions from other studies [25,30,31]. However, one study showed a striking effect in patient under age 65 with mixed conditions requiring rehabilitation [32]. Apart from age and functional impairment level, other factors affecting the duration of stay of stroke patients in hospital include delay in provision of equipment and home adaptation, waiting for private home placement (negative impact), and frequency of consultant ward round ≥ 1 per week (positive impact) [20], and also better continuity of care by combining acute and rehabilitative services [33]. Clearly the type and accessibility of community support are also likely to influence duration of stay. In this study, in term of contact hours with allied health staff, there were no major differences between the three institutions. All had a main physiotherapy and occupational therapy department where patients received treatment in for about one hour in each; however it was not possible to establish the exact staff to patient ratio. Some difference existed in the organization of rehabilitation services between the institutions. In institution A, rehabilitation within the wards was encouraged, and nursing staff, personal care workers as well as relatives were considered part of the team. Each patient had a label on the wall by the head of the bed to state the level of functioning and each ward had a patient functional summary chart so that care by ward staff would be appropriate to a particular level of function. In this way patients were encouraged to do as much as possible. Furthermore, there were weekly multidisciplinary ward rounds conducted by a consultant in geriatric/rehabilitation medicine or equivalent, including nurses, allied health staff and social workers. Service from a clinical psychologist, psychiatrists and speech therapist were also readily available. At the same time, a pre-discharge plan was formulated early on after admission, where close liaison with the family regarding progress and likely discharge ability was maintained. Social workers were involved early in the discussion if community support or institutional care would likely be needed on discharge. There was also a Geriatric Day Hospital on the site of Hospital A, enabling early discharge with subsequent continuing rehabilitation after discharge either to home or a long term residential care institution. There was a seamless transition from in patient to day patient services, and staff rotate or work simultaneously between inpatient and day patient settings. Thus Hospital A had a different process of care compared with the other institution, even though the structure of care may be similar. Elsewhere, it has been reported that better process of care, which depended on system organization, staff expertise and technological sophistication, was associated with better 6 month functional outcomes [33,34]. Interestingly the finding with respect to rehabilitation is different to that for mortality for stroke patients, where the best outcome (lowest mortality) is observed for hospital with the highest volume of patients [35]. In our study, hospital A had the lowest volume.

Within hospital A over a ten year period where the process of care remained essentially unchanged, we were able to observe the impact of staff numbers on outcome, as a result of cost cutting measures. From the mid 1990s, Hospital A began to experience progressive reduction in staff numbers, in a series of productivity gain initiatives. In order to maintain the process of care, two major reorganizations were carried out to cope with reduction in staff numbers. Patients were grouped so that those who were very dependent with multiple comorbidities with little rehabilitation potential received reduced rehabilitation service, so that those for stroke patients could be maintained (1999). In addition, one ward was closed (2003). While these measures produced a transient rise in FIMEG, the improvement could not be sustained. These observations suggest that although a good process of care may be in place, beneficial outcomes could be eroded by relentless reduction in staff number, and that an optimum number of staff in relation to best outcomes could be defined. It further highlights the need for health managers to consider outcome, as opposed to process measure, in making resource allocation decision.

This study also highlights the need for community services for stroke patients discharged from hospital. Given that the duration for potential maximal recovery far exceeds the duration of stay, the development of easily accessible community rehabilitation and home care support is of vital importance. If such services are not well developed or absent, then the continuing care pathway for stroke patients will be long term residential care. In the latter setting, not only is there little provision for continuing active rehabilitation to maximize an individual's potential; rather, those who had achieved a certain level start an inevitable decline. Currently over 50% of residents of institutional care have a diagnosis of stroke [36], and the waiting list for government subvented homes is in terms of 3 years or more.

There are limitations in this study, in that it is an analysis of retrospective data. There is no detailed documentation of individual patients contact time with therapists. There may be other confounding factors which were not included in the database and therefore could not be analyzed, in the inter-institutional and longitudinal comparisons. The inter institutional process of care comparison was not very detailed. No distinction was made between recurrent and first stroke patients. Nevertheless, the data analysis revealed variations in FIMEG, a stroke outcome measure, raising important questions in the current optimal care process and staff numbers.

## Conclusion

The finding suggest that interdisciplinary care with seamless interface with community may result in better outcome from the management point of view (FIMEG), but that this requires a level of staffing below which the outcome will be adversely affected. Finally the study highlights the need for well developed community services to maximize the rehabilitation potential for all stroke patients.

## Competing interests

The author(s) declare that they have no competing interests.

## Authors' contributions

JW conceived the idea of the paper, directed data synthesis and analysis, and wrote the manuscript. SYC and MWCS were responsible for the data collection and data synthesis, and participated in discussions of the manuscript. EW carried out data analysis. YPMC provided the nursing data and contributed the discussions of the results.

## Pre-publication history

The pre-publication history for this paper can be accessed here:


